# Mitral Annular Calcification-Related Valvular Disease: A Challenging Entity

**DOI:** 10.3390/jcm13030896

**Published:** 2024-02-03

**Authors:** Abdulrahman S. Museedi, Thierry H. Le Jemtel

**Affiliations:** Section of Cardiology, John W. Deming Department of Medicine, Tulane University School of Medicine, Tulane University Heart and Vascular Institute, New Orleans, LA 70112, USA; amuseedi@tulane.edu

**Keywords:** MAC, degenerative mitral valve, mitral valve annulus, mitral regurgitation, mitral stenosis, MAC-related mitral valve disease, mitral annular calcification

## Abstract

Mitral valve annular calcification-related valvular disease is increasingly common due to the rising prevalence of age-related mitral annular calcifications. Mitral annular calcification alters the structure and function of the mitral valve annulus, which in turn causes mitral valve regurgitation, stenosis, or both. As it frequently coexists with comorbid conditions and overlapping symptoms, mitral annular calcification-related valvular disease poses significant diagnostic and therapeutic challenges. For instance, left ventricular diastolic dysfunction hinders the assessment of mitral valvular disease. Detection of mitral annular calcifications and assessment of related mitral valve disease hinge on two-dimensional echocardiography. Comprehensive assessment of mitral annular calcifications and related mitral valve disease may require multidetector computed tomography and three-dimensional echocardiography. Invasive hemodynamic testing with exercise helps identify the cause of symptoms in patients with comorbid conditions, and transcatheter interventions have emerged as a viable therapeutic option for older patients. After an outline of the normal mitral annulus, we examine how mitral annular calcifications lead to mitral valve disease and how to accurately assess mitral regurgitation and stenosis. Lastly, we review surgical and transcatheter approaches to the management of mitral annular calcification-related mitral valve regurgitation, stenosis, or both.

## 1. Introduction

The prevalence of mitral annular calcification (MAC) and related valvular disease is increasing as the population ages. The prevalence of MAC varies from 5 to 42%. The age of study participants and the imaging modality used for MAC detection account for the wide variance [1,2,3]. Two population-based studies reported a prevalence of 2.2% and 6.6% for MAC-related mitral stenosis (MS) and 11.9% and 9.5% for significant MAC-related mitral regurgitation (MR) [4,5]. Among patients with mitral valve disease (MVD), patients with MAC-related MVD have the lowest survival rates. The one-year survival rate post-diagnosis is 76% in patients with MAC-related MVD and 86% in patients with other MVD [5]. 

The development of MAC is viewed as a degenerative aging process. However, besides aging, the following mechanisms contribute to the development and progression of MAC [6]:

1. Atherosclerotic process: There is a well-established correlation between MAC and vascular diseases, such as coronary artery disease [7,8], carotid artery disease, and strokes [1,2]. Hence, MAC and atherosclerosis may share common mechanisms.
2. Calcium phosphate metabolism: A dysregulated calcium phosphate metabolism in patients with chronic kidney disease may result in calcium deposition, contributing to MAC [9].
3. Mechanical stress: Increased stress on the mitral annulus, mitral valve (MV), and MV apparatus is often due to left ventricular (LV) hypertrophy (LVH) and elevated LV pressure. Elderly patients with MAC commonly exhibit abnormal LV diastolic function, left atrial enlargement, and compromised left atrium (LA) reservoir strain, thereby highlighting the high prevalence of MAC in patients with heart failure and preserved ejection fraction (HFpEF) [10].
4. Inflammation: There is growing evidence linking MAC to inflammatory processes, as illustrated by elevated inflammatory markers in patients with MAC [11]. Imaging with F18-fluorodeoxyglucose (FDG) reveals increased FDG uptake in patients with MAC [12].

Epicardial adipose tissue secretes inflammatory mediators and cytokines [13]. Likely due to heightened inflammation, the thickness of epicardial adipose tissue is an independent predictor of the severity of MAC [14].

## 2. Mitral Valve Physiology and Mechanisms of Related Mitral Valve Disease

The mitral valve annulus serves as a boundary between the LA and the LV. The mitral annulus has an anterior and posterior segment. The anterior segment connects the aortic root to the anterior leaflet of the mitral valve and thereby forms the aorto-mitral curtain. The anterior and posterior segments act as anchoring points for the respective MV leaflets [15,16]. 

The annulus possesses a dynamic, non-planar configuration that plays a crucial role in the MV [15,16]. 

**Figure 1:**
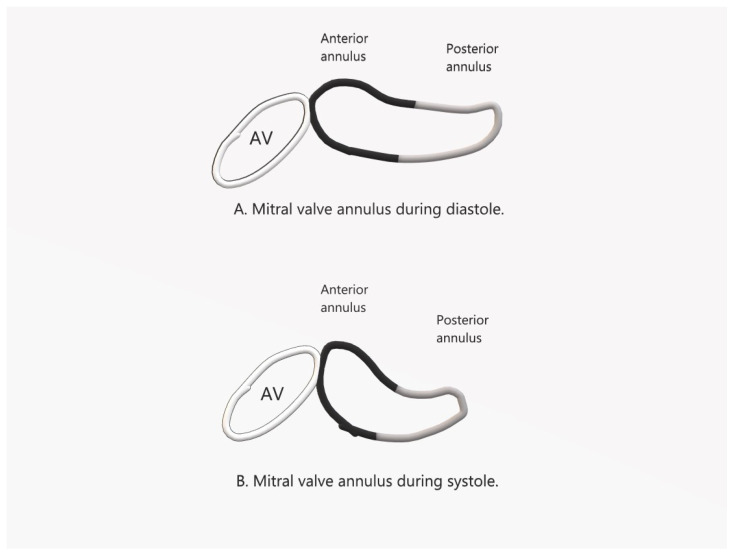
Illustration of the mitral valve annulus during diastole and systole demonstrating the increase in the anterior horn saddle height, folding along the inter-commissural diameter, and reduction in mitral valve area during systole. AV: aortic valve.

Characteristically, the annulus adopts a saddle-shaped form with distinctive anterior and posterior horns. During the systolic phase of the LV, the annulus moves downward. However, as the downward motion of the posterior horn is greater than that of the anterior horn, the annulus folds along the inter-commissural diameter, bringing the anterior annulus closer to the posterior annulus during LV systole [16] (Figure 1). The differential motion accentuates the saddle shape during LV systole (deepening of the saddle height), thereby promoting valve coaptation and deterring MV regurgitation. Additionally, the differential downward motion minimizes stress on the MV leaflets by reducing the mitral valve area by 20–42%, allowing a more resilient MV to withstand elevated LV systolic pressures [15,16,17].

Moderate-to-severe MAC affects the downward motion of the annulus. Compared to the normal annulus, the anteroposterior (AP) diameter is larger in diastole and decreases less during systole. Furthermore, the annulus flattens during LV systole with a lesser deepening of the saddle height in patients with MAC [18].

The greater abundance of MAC in the posterior annulus than in the anterior annulus [19] is presumably related to the high fat content of the posterior leaflet. MAC can expand to the leaflets, papillary muscle, and LV wall [20]. 

**Figure 2:**
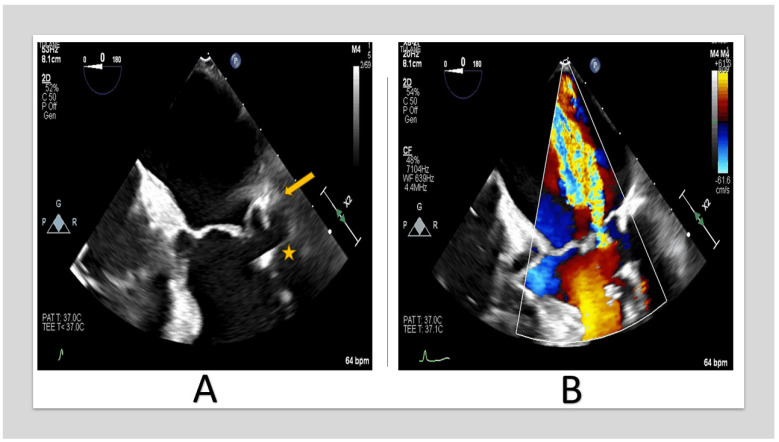
TEE images of 67-year-old woman with severe MR. (**A**) shows posterior annulus calcification (arrow) with sub-valvular apparatus extension (star. (**B**) shows posteriorly directed MR jet due to posterior leaflet restriction. (TEE: transesophageal echocardiography, MR: mitral regurgitation).

The following mechanisms underlie MVD development in MAC:

Extension of the posterior annulus calcifications to the posterior leaflet and sub-valvular apparatus resulting in restricted motion of the posterior leaflet and, therefore, lack of coaptation [21] (Figure 2).

Abundant MAC results in impaired dynamic function of the annulus, which is less saddled during LV systole and shows less leaflet coaptation [21].

Sub-valvular calcifications may push the posterior leaflet toward the atrium, causing malcoaptation [16]; additionally, the latter mechanism might increase the tension on calcified chordae and lead to chordal rupture and flail leaflet [20].

**Figure 3:**
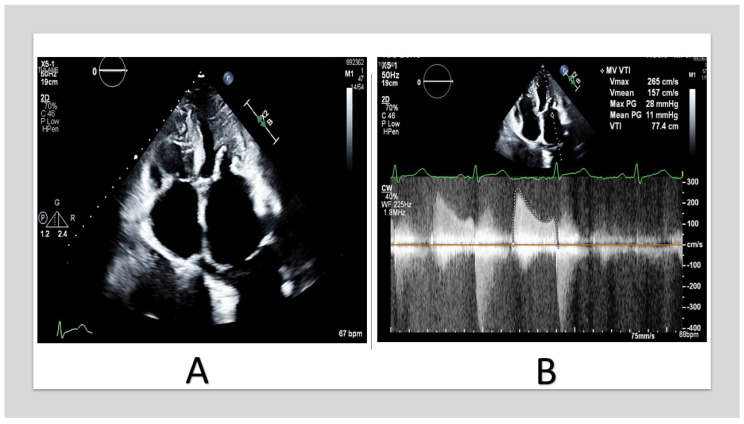
TTE of 57_year_old woman with end-stage renal disease. (**A**) shows severe MAC involving the anterior and the posterior annulus with extension to the leaflets and the chordae. (**B**) shows mitral inflow continuous-wave Doppler with mean gradient of 11 mmHg. (TTE: transthoracic echocardiography, MAC: mitral annular calcification).

**Figure 4:**
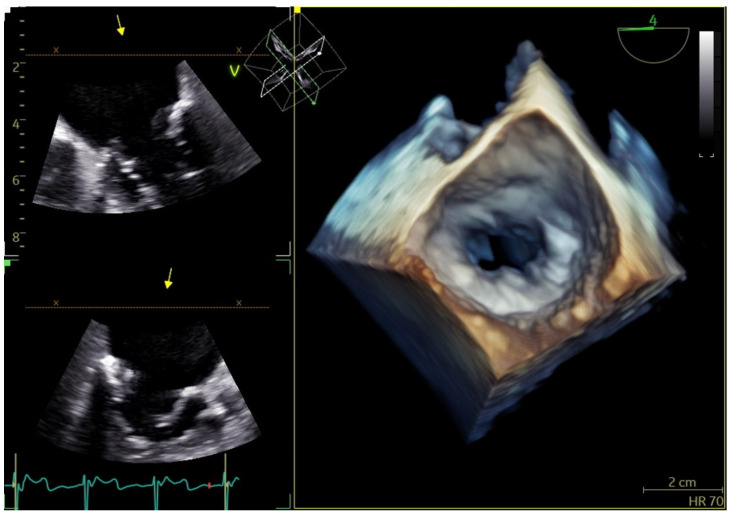
Three-dimensional TEE: severe MAC involves >270° of the annulus and results in MS due to displacement of the annulus inward. (TEE: transesophageal echocardiography, MAC: mitral annular calcification, MS: mitral stenosis).

On the other hand, MS is primarily due to the annular calcium shelf displacing the valve annulus inward along with calcifications extending toward the base of the leaflets, resulting in inflow obstruction [21] (Figure 3 and Figure 4). Involvement of the anterior annulus is a key contributor to the development of MS [19]. Unlike rheumatic MS (RMS), where the obstruction is at the tip of the leaflets, the obstruction is at the annulus base of the leaflets in degenerative MS. Furthermore, the calcification is non-planar and results in tubular inflow, unlike the funnel shape in RMS [19]. 

### 2.1. MAC Natural History

Over a 10-year follow-up, 22 and 4% of patients with mild MAC progressed to severe MAC and MAC-related MVD, respectively [22]. Among the patients with moderate MAC, 71 and 23% progressed to severe MAC and MAC-related MVD, respectively [22]. Within 18–36 months of the initial diagnosis, nearly one-third of patients with MAC progress, as evidenced by a rising trans-mitral mean pressure gradient and MAC angle in the parasternal shortaxis view by 2D TTE [23]. Patients with progressive MAC had smaller LV end systolic dimensions and higher ejection fraction, systolic blood pressure and pulse pressure than those with stable MAC [23]. Over a median follow-up of 39.2 months, patients with progressive MAC had worse clinical outcomes than patients with stable MAC [23]. Control of hemodynamic stress and comorbidities may delay progression of MAC. 

Rarely, MAC evolves into a caseous form. While the relationship between caseous MAC and MVD remains unclear, it introduces challenges to procedural planning for transcatheter valve interventions, coupled with an increased risk of stroke [24].

### 2.2. MAC Assessment and Grading

Two-dimensional (2D) echocardiography is the core imaging modality to detail MAC and assess MAC amount and impact on MV function. The thickness of calcifications from the leading edge of the anterior annulus to the trailing edge of the posterior leaflet and the extent of circumferential calcification helps us to appraise the amount of MAC [2,25]. Two-dimensional echocardiography has notable limitations for MAC assessment and grading. It cannot distinguish between fibrosis and calcification, and MAC can cast shadows that obscure underlying structures. Transesophageal echocardiography (TEE), particularly with three-dimensional imaging, offers a superior assessment over transthoracic echocardiography (TTE) due to enhanced visualization [26]. 

Three-dimensional (3D) TEE is as accurate as multidetector computed tomography (MDCT) in assessing mitral valve geometry [27], but MDCT is superior for MAC assessment [28]. However, 3D TEE with maximum intensity projection enhances calcification evaluation by making the calcified spots more irregular/prominent and providing the echocardiographer with improved insights into calcification characteristics [29,30].

**Figure 5:**
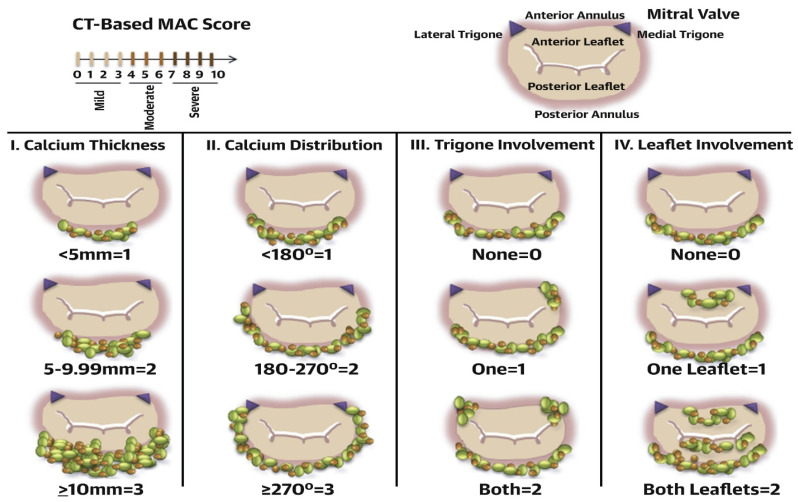
Elements used in the MAC score and their corresponding points. Average annulus calcium thickness (<5 mm = 1 point, 5 to 9.9 mm = 2 points, ≥10 mm = 3 points); calcium distribution in annulus circumference (<180° = 1 point, 180° to 270° = 2, ≥270° = 3); trigone calcification (none = 0, anterolateral = 1, posteromedial = 1); and mitral leaflet calcification (none = 0, anterior = 1, posterior = 1). A severity grade is assigned based on total points accumulated as follows: mild MAC = 3 points or less, moderate MAC = 4 to 6 points, and severe MAC ≥ 7 points. CT = computed tomography; MAC = mitral annular calcification. Reproduced with permission from Guerrero et al [31]., A Cardiac Computed Tomography–Based Score to Categorize Mitral Annular Calcification Severity and Predict Valve Embolization, *JACC*: *Cardiovascular Imaging*, Volume 13, Issue 9, September 2020, Pages 1945–1957. Copyright [2020] [American College of Cardiology Foundation].

MDCT has a high spatial resolution that enables MAC grading. Offering a superior visualization of MAC, MDCT provides a comprehensive evaluation of MAC and supports a novel grading system based on the circumference and thickness of MAC as well as the involvement of leaflets and trigones (Figure 5) [31]. The novel grading system may predict the risk of valve embolization during transcatheter valve replacement procedures. In fact, the Heart Valve Collaboratory [26] integrated the MDCT score into clinical, echocardiographic, and anatomical data to refine the assessment of patient suitability for potential interventions.

### 2.3. MAC-Related Mitral Valve Disease Assessment 

While MDCT is superior to 2D echocardiography for grading the amount of MAC, 2D echocardiography is the core imaging modality for assessment of MAC-related MVD. The definition of severe MAC-related MVD includes the presence of moderate-to-severe MR or severe MS (valve area < 1.5 cm^2^) [21]. The coexistence of MR and MS, which is common, adds complexity to the evaluation of MAC-related MVD [21]. Trans-mitral pressure gradient (TMG) closely correlates with mortality in patients with MAC-related MVD [32,33].

An updated definition of severe MAC-related MVD includes severe MS (valve area < 1.5 cm^2^), moderate-to-severe MR or TMG > 8–10 mmHg along with the presence of severe stenosis and regurgitation [21]. 

MAC-related MVD frequently affects elderly patients who have comorbid conditions like HFpEF, hypertension, aortic stenosis (AS), LVH, and depressed LV compliance [34] that lead to LA enlargement, reduced LA compliance, and abnormal LA–LV coupling [35]. Patients with reduced LA compliance and advanced LV diastolic dysfunction have a high LA V wave followed by a steep y descend due to rapid LV filling of a poorly compliant LV (high E wave velocity with short deceleration time on echocardiography) that heightens TMG in the early phase of diastole [34]. Thus, any degree of superimposed degenerative MS accentuates the TMG. In contrast to degenerative MS, the LV compliance is normal in RMS [36]; the y descend is less steep and the TMG closely reflects stenosis in RMS MS [34]. Chiefly, HFpEF may underlie the increased mortality of patients with high TMG and MAC-related MVD.

Ascertaining whether exertional symptoms are due to MAC-related MVD or HFpEF is a clinical challenge. Further, the presence of MAC complicates the assessment of LV diastolic function by 2D echocardiography [37]. By monitoring LV diastolic pressure and LA pressure during exercise, invasive hemodynamic testing may help to determine whether MAC-related MVD or LV diastolic dysfunction is primarily responsible for exertional symptoms in very old patients with HFpEF and MAC [34]. 

On the other hand, severe MS disproportionately affects women more than men [19,32]. With a lower body surface area and a smaller LV cavity and thereby a lower stroke volume than men, women may have severe MS and low TMG [19]. 

### 2.4. MS Assessment 

Assessing the severity of MAC-related MS is a significant hurdle due to the lack of reliable measurement methods. The TMG may be misleading, as we discussed in the previous section.

The continuity equation is often used to evaluate MV area. However, it cannot be used in patients with mitral regurgitation (MR) or aortic insufficiency (AI) [38]. The pressure half-time method is validated and shows a strong correlation with MV area, as measured by the Gorlin equation [39] in patients with RMS who have normal left atrial and ventricular compliance. The pressure half-time method can lead to overestimation of MV area in patients with MAC and MAC-related MVD who have depressed LV and LA compliance [40].

The proximal isovelocity surface area (PISA) method is problematic in MAC-related MS. The valve’s tubular geometry hinders the formation of a hemispherical flow convergence region that the PISA method assumes.

**Figure 6:**
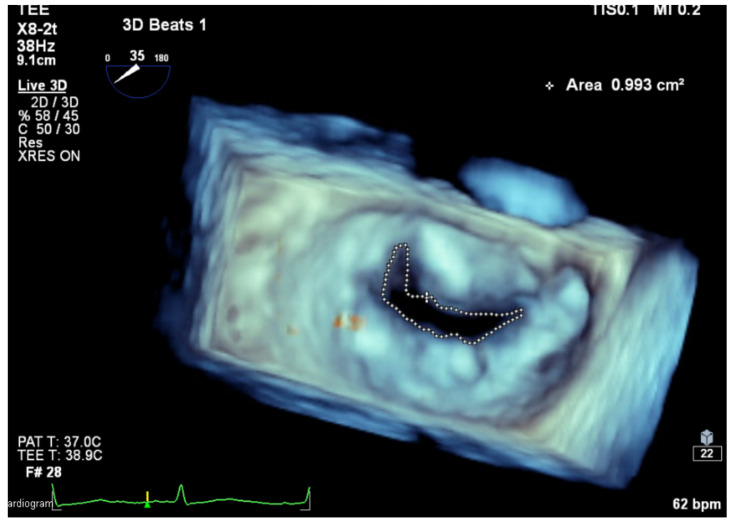
MVA measurement using 3D TEE planimetry. (MVA: mitral valve area, 3D TEE: three-dimensional transesophageal echocardiography).

Planimetry by 2D echocardiography accurately evaluates the severity of RMS [41], where the mitral valve typically presents as a funnel shape, allowing for precise measurement at the leaflet tips. In contrast, the tubular and non-planar geometry seen in MAC-related MS thwarts the accuracy of planimetry by 2D echocardiography. Planimetry by three-dimensional (3D) echocardiography is as accurate as volumetric assessment of MAC-related MS independent of MR or AI [42] (see Table 1 and Figure 6). In patients with MAC-related MVD, the role of MDCT is limited to MV anatomy and planimetry of MV area.

### 2.5. MR Assessment 

Shadowing from MAC affects the assessment of MAC-related MR by transthoracic 2D echocardiography. It impairs visualization of the vena contracta and jet area and the accuracy of color Doppler and continuous wave (CW) assessments. Thus, transesophageal 2D echocardiography (TEE) is a superior option in patients with MAC, as its position beyond the area of calcification minimizes the impact of shadowing [38].

However, the quantitative methods for evaluating degenerative MR have their own limitations. The PISA method is a widely recognized method for the measurement of the effective regurgitant orifice area (EROA) and regurgitant volume (Rvol) [43]. In MAC-related MR, where jets are often eccentric, the PISA method does not form a perfect hemisphere and may overestimate the EROA and, subsequently, the Rvol. Furthermore, the use of the continuity equation for MR assessment can be compromised by concurrent AI—a not uncommon finding in patients with aortic valve calcification. The accuracy of the continuity equation also depends on precise measurement of the LV outflow tract (LVOT) diameter, a common source of error.

**Figure 7:**
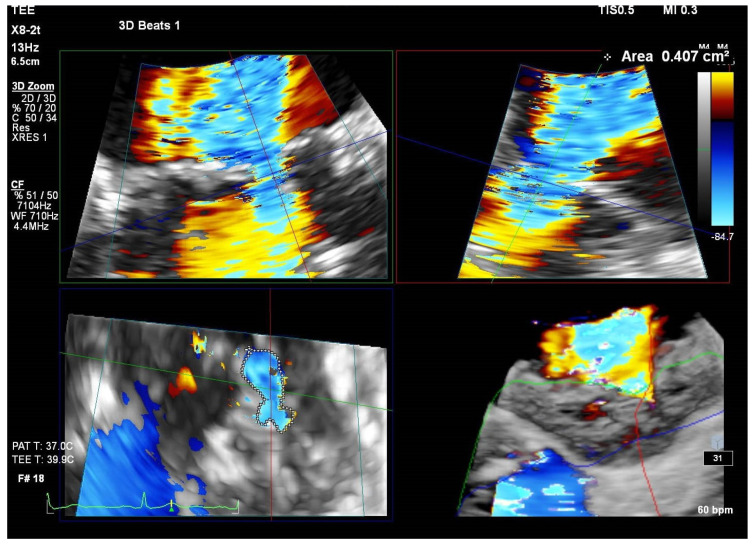
Measurement of vena contracta area by 3D TEE. (3D TEE: three-dimensional transesophageal echocardiography).

Three-dimensional echocardiography is a promising tool for the evaluation of MAC-related MR. Specifically, 3D vena contracta area obtained via TEE with 3D color Doppler provides a more accurate appraisal of MAC-related MR severity. A study by Goebel et al. [44] indicates that 3D vena contracta area (see Figure 7) correlates more closely with Rvol than the PISA method. The latter tends to overestimate EROA, leading to a potential misclassification of moderate MR as severe (see Table 2).

### 2.6. MAC and Aortic Stenosis

The frequent occurrence of MAC in patients with calcific AS suggests that MAC and AS share some underlying mechanisms [45]. The prevalence of MAC, severe MAC, and MAC-related MVD in individuals with severe calcific AS who underwent transcatheter aortic valve replacement (TAVR) was 43%, 10%, and 6.8%, respectively [46]. The presence of MAC might increase cardiovascular mortality after TAVR [46]. However, in agreement with Okuno et al. [47], the sole presence of MAC, even in its severe form, did not significantly increase all-cause mortality during the 30-day and 1-year follow-up periods. In contrast, several studies reported that the presence of MAC-related MVD was associated with higher mortality rates at both the 30-day and 1-year marks [46,47,48,49,50,51,52,53].

MR was expected to improve after TAVR due to a reduction in LV pressure, reverse remodeling that results in a smaller annulus, and LVEF improvement in patients with low-flow, low-gradient AS. However, several studies have shown that MAC-related MR is unlikely to improve after TAVR [52,54,55]. The lack of improvement in MR post-TAVR worsens outcomes post-TAVR [56]

The common coexistence of severe calcific AS and MAC-related MVD begets a challenging multivalvular condition. Typically, patients with calcific AS and MAC-related MVD have elevated surgical and anatomical risks and are less amenable to conventional surgical interventions. Currently, most reports of transcatheter valve interventions target both valves simultaneously [57,58]. 

### 2.7. Management

The presence of MAC is surgically challenging. Any amount of MAC increases operative mortality and complications [59]. Patients with MAC-related MVD confront two major risks: elevated surgical risk attributed to very old age and multiple comorbidities and anatomical risk determined by the amount of MAC. Both risks guide the selection of interventions [60]. 

Surgical techniques can be broadly categorized into two groups: MV surgery with MAC resection and annulus reconstruction and MV surgery without MAC resection [61], also termed as resect vs. respect [62]. The resection technique carries risks of atrioventricular groove dissociation, LV perforation, and injury to the left circumflex artery. Conversely, the respect approach presents increased risk of paravalvular leak due to suboptimal suture anchoring to the calcified annulus and the tendency to use a smaller valve with the risk of valve prosthesis mismatch [61,62]. 

In a systematic review [63], 15 surgical studies reported wide ranges of mortalities at 30 days, 1 year, and 5 years: 0% to 27.3% (median 6.3%), 0–17% (median 15.8%), and 0–68.6% (median 38.8%), respectively. Variances in mortality rates are likely attributable to broad surgical and anatomical risks that may have been underreported in some studies. Whether the minimally invasive surgical approaches [64,65,66] can benefit patients with MAC-related MR is unclear. 

For patients with low surgical risk and anatomically feasible conditions, the surgical option remains the optimal choice for managing degenerative MVD.

For patients with very high surgical or anatomical risk, or both, the transcatheter approach is being considered increasingly often. The first case of human transcatheter mitral valve replacement (TMVR) was reported in 2009 by Cheung et al. [67] using the transapical approach for the valve-in-valve (ViV) TMVR. 

Commonly, TMVR is performed using a transfemoral transeptal approach with a balloon-expandable valve (SAPIEN valve from Edwards Lifesciences LLC) originally designed for transcatheter aortic valve replacement (TAVR) [68]. Transapical or direct transatrial approaches have also been used [68]. 

The median age was 75 years in a systematic review of 13 studies encompassing 354 patients who underwent transseptal or transapical TMVR [63]. The technical success rate for transeptal TMVR was 75%, with LV outflow tract (LVOT) obstruction occurring in 11.2%. The median in-hospital, 30-day, and 1-year mortality rates for TMVR in patients with MAC were 16.7%, 22.7%, and 43%, respectively.

The mean age was 79 years and the New York Heart Association (NYHA) functional class was III-IV in an early cohort of 12 patients who underwent TMVR for MAC-related MVD [69]. In total, 67% of patients had mitral stenosis and 25% had mixed MAC-related MVD. One patient developed LVOT obstruction and later died. Three patients displayed valve migration, one with complete embolization to LA requiring bailout surgery and two with slight valve migration resulting in severe paravalvular leak. Survival rates at 30 days and 1 year were 83% and 57%, respectively, with 9 out of 10 surviving patients reporting improved exercise tolerance at 30 days and 3 out of 4 patients reporting improved symptoms at 1 year.

Although the survival of patients with MAC was initially poor after TMVR, selection and procedural insights have been gained. Patients with a modest amount of MAC are prone to valve embolization and migration due to insufficient calcium for anchoring. Unexpectedly, a sizeable amount of MAC proved favorable for procedural success. Identification of patients prone to LVOT obstruction helped reduce procedural mortality. 

The two largest cohorts of TMVR in MAC, the MAC global registry (*n* = 106) [70] and STS/ACC/TVT registry (*n* = 100) [71], reported LVOT obstructions in 11.2% and 10% of patients, respectively. Strategies were devised to mitigate LVOT obstruction and reduce procedural mortality. The first strategy, reported in the MITRAL trial [72], involved preemptive alcohol septal ablation 3–4 weeks before TMVR. The strategy was carried out in seven patients who were identified as being at high risk of LVOT obstruction. It was technically successful, and all seven patients survived the 30-day period. The second strategy, tested in a small single-arm trial, included 30 patients with indication for TMVR in MAC or annuloplasty ring. The strategy involved transcatheter intentional laceration of the anterior mitral valve leaflet (LAMPOON) [73] and resulted in an 87% survival rate at 30 days post-op in patients with MAC. The strategy intended to copycat the anterior leaflet resection during surgical MVR. In patients with MAC, TMVR remains a very high-risk intervention that may benefit highly selected patients who failed optimal medical therapy of co-existent conditions like HFpEF and COPD. Further, TMVR should be performed in experienced centers for patients with favorable anatomy.

Experience with transcatheter edge-to-edge repair (TEER) is limited in patients with MAC-related MR, as severe MAC was one of the exclusion criteria in the EVERESTII trial [74]. Nevertheless, TEER appears safe in selected patients with moderate-to-severe MAC [75,76,77,78]. Patients with MAC-related MR and mitral valve area < 4cm^2^, calcification extending to the margin of the leaflets and coexisting MS are not candidates for TEER [76].

## 3. Conclusions

The management of MAC-related MVD poses a growing clinical challenge. Determining whether symptoms are mostly due to MVD is an arduous task in patients with coexisting conditions. In some clinical scenarios, invasive hemodynamic assessment with exercise may be the sole method for differentiation. Additionally, grading the severity of the valvular disease presents another challenge, as most echocardiography methods have limitations in assessing MAC-related MVD. Notably, 3D echocardiography, particularly with TEE, stands out as the most accurate means of grading severity.

Most patients with MAC-related MVD have high surgical risks due to the presence of multiple comorbidities coupled with the technical challenges associated with MAC. Consequently, TMVR in MAC has emerged as a viable therapeutic option. However, reducing the risk of LVOT obstruction requires further investigation. Lastly, TEER may be an option for patients with MAC-related MR who are free of MS with mitral valve area > 4 cm^2^ and margins free of calcification.

## Figures and Tables

**Table 1:** MAC-related MS assessment methods.

Method	Limitation
Continuity equation	Limited by the coexistence of MR or AI.
Pressure half-time	Limited by coexistence of abnormal LV and LA compliance.
PISA method	Limited by lack of hemisphere formation.
2D planimetry	Limited by the non-planar geometry.
3D planimetry	Has fewer limitations comparing with other methods and is the most accurate.

**Table 2:** MAC-related MR assessment methods.

Method	Limitations
Continuous-wave Doppler	Limited by shadow from calcification.
2D vena contracta	Limited by shadow from calcification.
PISA method	Limited by lack of hemisphere formation.
Continuity equation	Limited by the coexistence of AI.
3D vena contracta	Fewer limitations. Most accurate echocardiographic method.
